# Supplementary material for: The inner junction complex of the cilia is an interaction hub that involves tubulin post-translational modifications
Source: eLife. 2020 Jan 17;9:e52760. doi: 10.7554/eLife.52760 (PMC6994238; doi:10.7554/eLife.52760)
Supplement: Supplementary file 1. [file elife-52760-supp1.docx]

**SUPPLEMENTARY FILE 1**

**Supplementary Table 1:** Significantly reduced or missing proteins in FAP52 compared to WT using relative mass spectrometry quantification.

| Names | Uniprot ID | **WT**  exclusive unique peptide counts (quantitative values after normalization) | **FAP52**  exclusive unique peptide counts (quantitative values after normalization) | **FAP52/WT ratio**  (quantitative values were used) | **p-values**  **(WT vs FAP52)** | **Log2(Fold Change *(*FAP52/WT))** |
| --- | --- | --- | --- | --- | --- | --- |
| **FAP20** | **A8IU92** | **14, 10, 12 (27, 19, 33)** | **14, 12, 13 (33, 17, 18)** | **0.86** | **0.65** | **-0.31** |
| **FAP45** | **A8I9E8** | **31, 27, 12 (60, 37, 33)** | **31, 30, 30 (48, 43, 40)** | **1.01** | **0.96** | **0.016** |
| **PACRG** | **A8I2Z6** | **13, 9, 10 (41, 26, 48)** | **15, 13, 13 (70, 42, 38)** | **1.30** | **0.38** | **0.38** |
| **Tektin** | **A8J8F6** | **20, 22, 14 (60, 61, 52)** | **29, 24 24 (74, 45, 43)** | **0.88** | **0.74** | **-0.096** |
| ARL3 | A8ISN6 | 2, 2, 1 (2, 1, 2) | 0, 0, 0 (0, 0, 0) | 0.0 | 0.0013 | Missing |
| CHLREDRAFT_171815 | A8HQQ4 | 2, 6, 1 (2, 5, 2) | 0, 0, 0 (0, 0, 0) | 0.0 | 0.035 | Missing |
| CHLREDRAFT_156073 | A8I1U2 | 1, 1, 1 (2, 1, 2) | 0, 0, 0 (0, 0, 0) | 0.0 | 0.024 | Missing |
| FAP276 | A8J9P2 | 3, 3, 2 (8 ,7, 14) | 0, 0, 0 (0, 0, 0) | 0.0 | 0.015 | Missing |
| CFAP52 | A8ILK1 | 27, 21, 15 (59, 73, 105) | 0, 0, 0 (0, 0, 0) | 0.0 | 0.0046 | Missing |
| FAP36 | A8IZX7 | 3, 3, 1 (3, 3, 2) | 0, 0, 0 (0, 0, 0) | 0.0 | 0.0023 | Missing |
| CrCDPK1 | A8IHF4 | 3, 5, 2 (3, 4, 4) | 0, 0, 0 (0, 0, 0) | 0.0 | <0.0001 | Missing |
| CHLREDRAFT_176830 | A8J922 | 2, 1, 2 (2, 1, 1) | 0, 0, 0 (0, 0, 0) | 0.0 | 0.024 | Missing |
| FAP173 | A8JAF7 | 3, 3, 1 (5, 3, 2) | 0, 0, 0 (0, 0, 0) | 0.0 | 0.012 | Missing |
| FAP29 | A8J3X6 | 2, 3, 2 (3, 3, 4) | 0, 0, 0 (0, 0, 0) | 0.0 | 0.00045 | Missing |
| CHLREDRAFT_181390 | A8JJY2 | 1, 1, 1 (1, 1, 2) | 0, 0, 0 (0, 0, 0) | 0.0 | 0.028 | Missing |
| ANK2 | A8HNK2 | 1, 2, 1 (1, 1, 2) | 0, 0, 0 (0, 0, 0) | 0.0 | 0,0041 | Missing |
| FAP5 | A8JAI0 | 11, 15, 7 (23, 22, 23) | 2, 0, 0 (1, 0, 0) | 0.014 | <0.0001 | -5.5 |
| FAP164 | A8JC79 | 4, 5, 1 (6, 4, 2) | 0, 0, 1 (0, 0, 0) | 0.0 | 0.022 | -5.1 |
| FAP288 | A8IJV3 | 13, 12, 9 (18, 14, 23) | 2, 1, 1 (1, 0, 0) | 0.018 | 0.0021 | -4.6 |
| CHLREDRAFT_177061 | A8J9A4 | 7, 7, 2 (8, 6, 4) | 0, 1, 1 (0, 0, 0) | 0.0 | 0.0071 | -4.5 |
| TEF20 | A8IL00 | 2, 1, 1 (2, 1, 2) | 0, 1, 0 (0, 0, 0) | 0.0 | 0.036 | -3.6 |
| CHLREDRAFT_191579 | A8J3S1 | 2, 4, 2 (3, 4, 4) | 1, 0 ,1 (1, 0 , 0) | 0.09 | 0.0003 | -3.4 |
| CHLREDRAFT_111330 | A8IAY6 | 2, 2, 1 (3, 1, 2) | 1, 0, 0 (1, 0, 0) | 0.16 | 0.03 | -3.2 |
| 14-3-3 | Q7X7A7 | 9, 9, 3 (15, 10, 6) | 5, 0, 1 (4, 0, 0) | 0.13 | 0.03 | -3.0 |
| Isocitrate lyase | A8J244 | 12, 8, 9 (23, 8, 19) | 6, 2, 2 (5, 1, 1) | 0.14 | 0.036 | -2.9 |
| CHLREDRAFT_141580 | A8I9N1 | 7, 11, 4 (13, 16, 17) | 3, 3, 3 (3, 2, 2) | 0.19 | 0.0006 | -2.7 |
| Elongation Factor 2 | A8JHX9 | 15,17,5 (27, 22, 10) | 3, 6, 6 (3, 3, 3) | 0.15 | 0.026 | -2.7 |
| CHLREDRAFT_189452 | A8IT59 | 4, 6, 1 (5, 5, 2) | 1, 1, 1 (1, 0, 1) | 0.17 | 0.026 | -2.7 |
| CHLREDRAFT_175290 | A8J364 | 4, 3, 2 (5, 3, 6) | 1, 2, 2 (1, 1, 1) | 0.21 | 0.014 | -2.6 |
| CHLREDRAFT_111269 | A8IBY2 | 1, 4, 1 (1, 3, 2) | 0, 2, 1 (0, 1, 0) | 0.17 | 0.045 | -2.4 |
| FAP138 | A8IUQ2 | 4, 6, 3 (8, 7, 6) | 2, 3, 3 (1, 1, 1) | 0.14 | 0.00046 | -2.4 |
| FAP31 | A8JDM7 | 6, 8, 4 (8, 7, 8) | 7, 0, 0 (5, 0, 0) | 0.21 | 0.024 | -2.2 |
| IFT80 | A8IXE2 | 4, 5, 1 (5, 4, 2) | 1, 1, 2 (1, 1, 1) | 0.27 | 0.022 | -2.2 |
| CHLREDRAFT_189792 | A8HPX1 | 16, 21, 6 (18, 17, 12) | 0, 11, 13 (0, 6, 5) | 0.23 | 0.0096 | -2.1 |
| CHLREDRAFT_206178 | A8IP72 | 17,  24, 10 (20, 19,23) | 0, 15, 18 (0, 7, 8) | 0.24 | 0.0053 | -2.0 |
| GSK3 | Q6IV67 | 4, 3, 2 (5, 2, 4) | 2, 2, 1 (1, 1, 0) | 0.18 | 0.030 | -1.8 |
| CHLREDRAFT_144025 | A8IF86 | 13, 27, 14 (16, 27, 35) | 14, 12, 13 (11, 7, 7) | 0.32 | 0.035 | -1.7 |
| FAP148 | A8IAT9 | 29, 41, 11 (44, 52, 27) | 14, 22, 26 (14, 12, 15) | 0.33 | 0.022 | -1.6 |
| FAP85 | A8J250 | 7, 11, 6 (14, 15, 19) | 8, 4, 4 (10, 3, 2) | 0.31 | 0.024 | -1.6 |
| Phototropin | A8IXU7 | 30, 22, 18 (56, 44, 64) | 25, 14, 17 (41,12, 13) | 0.40 | 0.041 | -1.3 |
| p38 | A4PET3 | 3, 8, 4 (7 ,7, 8) | 5, 3, 3 (5, 2, 1) | 0.36 | 0.019 | -1.3 |
| FAP39 | A8J0V2 | 6, 10, 4 (13, 11, 8) | 9, 5, 4 (7, 4, 3) | 0.42 | 0.022 | -1.1 |
